# Using a phiC31 “Disintegrase” to make new *attP* sites in the *Drosophila* genome at locations showing chromosomal position effects

**DOI:** 10.1371/journal.pone.0205538

**Published:** 2018-10-08

**Authors:** Mukesh Maharjan, Robert K. Maeda, François Karch, Craig M. Hart

**Affiliations:** 1 Department of Biological Sciences, Louisiana State University, Baton Rouge, Louisiana, United States of America; 2 Department of Genetics and Evolution, University of Geneva, Geneva, Switzerland; Texas A&M University College Station, UNITED STATES

## Abstract

An engineered phiC31 “Disintegrase” able to make an *attP* site in *Drosophila* out of an *attR*-*attL* pair is described. This was used to generate *attP* sites at genomic locations where a mini-*white* (mini-*w*) transgene was subject to chromosomal position effects (CPE). The first step was random genomic integration of a P-element-based transposon with an insulated mini-*w* transgene. We then removed the upstream insulator using FLP recombinase to detect CPE. Next mini-*w* and the downstream insulator were “dis-integrated” leaving behind an *attP* site. The location is marked by a *yellow*^*+*^ transgene that is flanked by *loxP* sites, so it can also be removed. Using this system, we generated 10 new *attP* landing platforms. Three of these showing strong activating CPE were selected for further analysis. We show that the *attP* sites are functional by integrating in plasmids with *attB* sites. The CPE is recapitulated and can be blocked by insulators. We show that a dimerized 215 bp fragment of the 500 bp BEAF-dependent scs’ insulator containing a high affinity BEAF binding site blocks the CPE, while a monomer of the sequence is less effective. This indicates that two BEAF binding sites make a stronger insulator than a single site. This system could be useful for generating *attP* sites at prescreened sites for other purposes, such as studying CPE in embryos or other tissues or for use with “trapped” enhancers of interest.

## Introduction

The use of transgenic organisms for purposes such as expressing normal or mutant proteins or studying potential regulatory elements is an important tool in basic and applied research. In *Drosophila*, the use of modified P-element DNA transposons has historically been the most common method for generating transgenic flies [1]. P-element integration into the genome is fairly random, so each integration event needs to be individually characterized as to which chromosome is affected and, if desired, exactly where the insertion occurred. In addition, different insertion events are subject to different chromosomal position effects (CPE) depending on local regulatory elements and chromatin states. Randomness can be advantageous for generating insertion mutations or enhancer-trapping, but can complicate the analysis of transgenes and regulatory elements. One solution is to use site-specific integration to standardize CPE. The integration mechanism of the bacteriophage phiC31 [2] has been adapted for this purpose in *Drosophila*, creating a powerful method for integrating different DNA sequences at the same genetic locus. This requires placing an *attP* integration site into the fly genome, an *attB* site into the plasmid containing the DNA to be integrated into the genome, and a source of the phiC31 Integrase [3–6]. Alternatively, cassette exchange can be done using a pair of *attP* and *attB* sites [7].

While many *attP* landing platforms exist, they are not always suitable. For instance, we are interested in chromatin domain insulator elements. One assay for insulator activity is their ability to shield a bracketed transgene from CPE, resulting in position-independent expression [8, 9]. However, when using random integration mediated by P-element insertion, this requires sampling multiple genomic locations so a statistically significant conclusion can be reached as to whether a test element has insulator activity or not. Ideally, ten or more genomic locations should be sampled for each test element. This assay would be simpler if all test constructs could be tested at the same chromosomal location. This requires that the location is subject to CPE, so there is an effect for the test element to block. We tested 13 *attP* sites available from the Bloomington *Drosophila* Stock Center (BDSC), and none were suitable for our purpose. They either did not exhibit CPE, or activation of the mini-*w* reporter gene was not blocked by an insulator. Therefore we wanted to generate new *attP* landing platforms that suit our needs. We wanted to place *attP* sites in genomic locations that show CPE activation of a mini-*w* reporter gene that can be blocked by an insulator element.

Here we describe the development of a “Drosophilized” transgene encoding a phiC31 “Disintegrase” (Dint) that can be used to collapse an *attR*-*attL* pair to an *attP* site. This is based on the pioneering work of the M.C. Smith lab [10], and is similar to a report that was published while this work was in progress [11]. This tool could be useful for purposes other than the one that we use it for here. We also developed a transposon in which an insulator element can be removed by FLP recombinase to test for CPE, and then the mini-*w* gene and insulator at its 3’ end can be “dis-integrated” using Dint to leave an *attP* site. Since this removes the mini-*w* marker gene, the transposon also has a *yellow*^*+*^ gene to mark the presence of the *attP* site in the remnant transposon. In addition, the *y*^*+*^ gene is bracketed by *loxP* sites so that it can be removed by Cre recombinase if desired. We use this system to generate several *attP* sites at locations that show varying degrees of CPE. We also show that CPE is still evident when we reintegrate insulated or uninsulated mini-*w* transgenes into 3 of the new *attP* platforms.

## Materials and methods

### Plasmids and DNA

All plasmids used here for testing insulator activity were made from pC4scs, a previously described derivative of pCaSpeR4 [12] containing a 990 bp scs insulator sequence inserted downstream of the mini-*w* gene [9]. A 50 bp *attB* site (CGGTGCGGGTGCCAGGGCGTGCCCTTGGGCTCCCCGGGCGCGTACTCCAC) was placed into this plasmid using XhoI and XbaI restriction sites to make pC4-attB-scs. The previously described 215 bp M fragment from scs’ including the high affinity BEAF binding site was inserted into the BamHI site of this plasmid as a monomer (M) or dimer (M2) [9]. The plasmid pRLY, which was used to generate new *attP* sites in the *Drosophila* genome, was constructed from pC4scs as follows. A 770 bp fragment containing a 50 bp *attR* site (GTAGTGCCCCAACTGGGGTAACCTTTGGGCTCCCCGGGCGCGTACTCCAC) followed by *FRT* sites bracketing the M2 insulator was placed upstream of mini-*w* using XhoI and EcoRI. The PstI site at the 3’ end of scs was used to insert a 50 bp *attL* site (CGGTGCGGGTGCCAGGGCGTGCCCTTGAGTTCTCTCAGTTGGGGGCGTAG) followed by *loxP* sites bracketing a 5 kb intronless *y*^*+*^ gene. The *y*^*+*^ gene [13] was from pC4-yellow [14]. This allows FLP recombinase-mediated removal of the M2 insulator to test for CPE on mini-*w* expression [15]. The mini-*w* gene and scs insulator can be removed by the phiC31 “Disintegrase”, hereafter referred to as Dint, leaving behind an *attP* site whose presence is marked by the *y*^*+*^ gene. If desired, the *y*^*+*^ gene can be removed by Cre recombinase.

The *Dint* transgene was made by overlap PCR using the nuclear-targeted *Drosophila* codon-optimized *integrase* as a template [3]. The following primers were used for the overlap PCR: NdeI dPhiC31-1S (AAAACATATGGACACGTATGCCGGTGC), dPhiC31-E449KS (CGACGCTTC GGCAAGCTCACTAAGGCGCCAGAGAAGTCCGGCG), dPhiC31-E449KAS (CGCCGGACTT CTCTGGCGCCTTAGTGAGCTTGCCGAAGCGTCG) and RI-nls-dPhiC31-1818AS (TTTT GAATTCTTACACCTTGCGCTTCTTCTTGGGGGCCGCTACGTCTTC-GGTGCC). After the overlap PCR reaction, the resulting PCR fragment was cloned into pGEMT-easy (Promega, Wisconsin, USA) and sequenced. To add the *nanos* promoter and UTRs, *Dint* was excised from pGEMT-easy with NdeI and EcoRI and subcloned into the pHSXnosN vector [16] cut with the same enzymes. A NotI fragment containing the *nanos* promoter, *nanos* 5’UTR, *Dint*, and *nanos* 3’UTR was then isolated from the resulting plasmid and cloned into a C4 yellow plasmid cut with NotI.

### Germline transformation and transposon hopping

Thirteen BDSC fly stocks with *attP* sites were tested for CPE. None were suitable (BDSC stock number and location in parentheses): ZH-22A (24481; 2L:22A2); VK37 (28472; 2L:22A3); ZH-51C (24482; 2R:51C1); ZH-51D (24483; 2R:51D9); ZH-58A (24484; 2R:58A3); VK31 (24870; 3L:62E1); VK33 (24871; 3L:65B2); ZH-68E (24485; 3L:68E1); ZH-86Fa (24486; 3R:86E18); ZH-86Fb (24749; 3R:86F8); ZH-96E (24487; 3R:96E10); VK20 (24867; 3R:99F8); ZH-102D (24488; 4:102F4). We designed the strategy described below to obtain suitable stocks.

Injections of pre-blastoderm embryos to generate transgenic flies were done by GenetiVision (Houston, TX) or in the Karch lab using standard techniques. The pRLY P-element plasmid was injected into a *y w* stock and resulted in one transgenic line, identified by eye color and body pigmentation. Three *attP* lines generated in this study were used to generate transgenic stocks using phiC31 Integrase [3, 4]. Two on the *X* chromosome were combined with *M{vas-int*.*B}ZH-102D* (from BDSC 23649), and one on chromosome *2* was combined with *y*^*1*^ *M{vas-int*.Dm}ZH-2A w^*^ (from BDSC 24486). The *Integrase* transgenes are marked with *3xP3-RFP*, so their presence can be confirmed by pink eye color in flies more than 4 days old. Stocks were sent to GenetiVision for injections with *attB* plasmids. The *Dint* construct was injected into a *y w* stock and the F1 were screened for inserts mapping to the X chromosome.

Injections with pRLY resulted in one fly stock with the transposon on chromosome *2*. To generate additional insertion sites, the *P{RLY}* transposon was placed over a *CyO* balancer chromosome and combined with *ry*^*506*^ *Sb*^*1*^ *P{ry*^*+t7*.*2*^ = *Delta2-3}99B* as a marked chromosome *3* transposase source (BDSC 3664). Among the hopped transposons recovered were two hops onto the *CyO* balancer. These were used in subsequent crosses to isolate additional hops, using the same transposase source. Chromosomes containing the new *P{RLY}* insertions were mapped using standard genetic methods with lab stocks of *y w* flies, *FM7/Df(1)JA52* flies, *CyO*/*wg*^*Sp-1*^ flies and *TM3*/*Scm*^*ET50*^ flies.

### Testing for chromosomal position effects (CPE)

To test for CPE, *P{RLY}* flies were crossed with *P{70FLP}10* flies (BDSC 6938) and third instar larvae were heat-shocked in a 37°C water bath for 1 hour to remove the M2 insulator. Resulting flies were crossed to flies with appropriate balancer chromosomes, and individual progeny were selected, made homozygous for the *P{RLY}* chromosome, and checked for removal of the M2 insulator by PCR. Homozygous *P{RLY}* and *P{RLYdelM2}* flies were crossed to *y w* flies, and eye color of 2 to 3 day old allelic heterozygous females was compared.

### Generating *attP* sites

Lines, especially those showing CPE, were selected for generating *attP* landing sites. Selected stocks were crossed to males with a *y w P{nos-Dint1*.*5 y} X* chromosome, and F_1_ female virgins were crossed to *y w* males with balancers as appropriate. Individual F_2_ *y*^*+*^ *w* males were crossed to *y w* females with balancers as appropriate, followed by crosses to assure removal of the *y w P{nos-Dint1*.*5 y}* chromosome and to make the *P{attP y}* chromosome homozygous. The presence of the *attP* site was verified by PCR and sequencing of the PCR product.

### Mapping P-element integration sites in genomic DNA

Mapping of integration sites of P-elements was done by performing TAIL-PCR [17], inverse PCR [18], or splinkerette PCR [19]. Products were sequenced and aligned to the genome using BLAST. Genomic primers were then designed and used with P-element primers to verify the genomic locations.

## Results

The use of site-specific integration allows different transgenes to be tested in the context of identical CPE. It also eliminates the need to map the site of transgene integration. The bacteriophage phiC31 Integrase together with *attP* sites has been adapted for this purpose in *Drosophila*, and is commonly used [3, 4]. In many cases it is desirable to minimize CPE. In our case, we are interested in chromatin domain insulator element function so we want a CPE that can be blocked by candidate insulator sequences. We tested 13 *attP* landing platforms available from the BDSC using a mini-*w* transgene with an scs insulator downstream and with or without the scs’-derived M2 insulator upstream [9]. None were suitable for our purpose. In order to generate *attP* landing sites at genomic locations subject to CPE that can be blocked by insulators, we developed two tools. One is a mutagenized phiC31 Integrase protein capable of mediating recombination between *attR* and *attL* sites to yield an *attP* site. The other is a P-element-based plasmid in which an insulator can be removed to test for CPE, and that has *attR* and *attL* sites to allow for creation of an *attP* site.

### Design of the phiC31 *Disintegrase* gene

**Fig 1:**
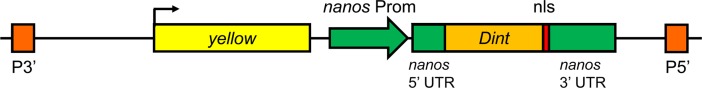
C4 yellow *Disintegrase* transposon. Shown in yellow is the *yellow* gene, which includes its promoter, 5’ UTR and 3’ UTR. Downstream of this is the *nanos* promoter with its 5’ and 3’ UTRs (green). Inserted between the *nanos* 5’ and 3’ UTRs is the *Disintegrase* sequence (light orange) fused to a nuclear localization sequence (nls in red). The 3’ and 5’ P inverted repeats are labeled in dark orange.

In 2008, the lab of M.C. Smith reported a phiC31 integrase mutant capable of catalyzing recombination between phiC31 *attR* and *attL* sites. This phiC31 integrase variant replaces a Glu at position 449 with a Lys [10]. To recreate this variant in the fruit fly, we replaced the equivalent Glu with a Lys in a nuclear-localized, *Drosophila* codon-optimized integrase [3] by overlap PCR. The gene for this mutant version the phiC31 integrase was then cloned into a P-element transposon vector where its expression and localization is controlled by the *nanos* promoter and UTRs. This results in a germline-expressing phiC31 E449K variant whose mRNA localizes to the posterior region of early embryos, where germ cell development initiates. We call this Drosophilized E449K version of the integrase, Disintegrase or Dint (Fig 1). We only determined the efficiency of dis-integration between an *attR* and *attL* site to yield an *attP* site for one fly line (see below), and found it to be over 90% efficient (129/136 flies).

### Design and use of a P-transposon to detect CPE

**Fig 2:**
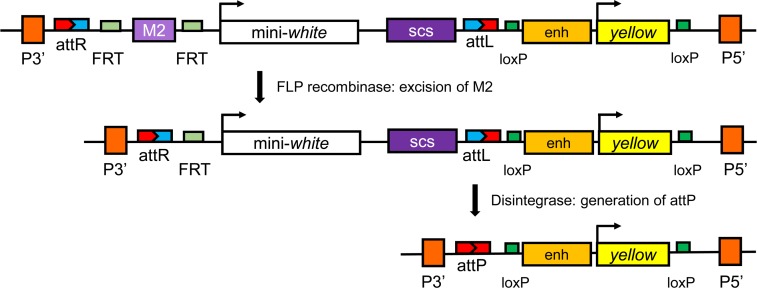
The *P{RLY}* transposon and the strategy for testing for CPE and making an *attP* landing site. The top shows the part of the pRLY plasmid between the P-element ends (orange rectangles). The mini-*w* gene is flanked by M2 (light purple rectangle) and scs (purple rectangle) insulator sequences. M2 is also flanked by *FRT* sites (light green rectangles) so it can be excised by FLP recombinase to test for CPE (middle of figure). The insulators and mini-*w* are flanked by *attR* and *attL* sites. The phiC31 Dint enzyme can excise these sequences, leaving an *attP* site (bottom of figure). Downstream is a transcription unit made of enhancers from the *y* gene upstream of a *y*^*+*^ cDNA to serve as a marker after mini-*w* is removed. There are *loxP* sites (green rectangles) flanking the *y*^*+*^ transgene so it can be removed by CRE recombinase.

We designed a P-element construct to allow us to identify genomic sites that exhibit CPE affecting *w* expression, and then to remove the *w*^*+*^ gene leaving behind an *attP* site to allow testing other candidate insulator sequences at the same location (Fig 2). We placed the BEAF-dependent scs’-derived M2 insulator [9], bracketed by *FRT* sites, upstream of a mini-*w* transgene with an scs insulator located downstream. An *attR* site was upstream, and an *attL* site was downstream of this assembly. Further downstream of this, the transposon has a *y*^*+*^ transgene bracketed by *loxP* sites to serve as a marker for the presence of the *attP* site after removal of mini-*w*.

**Fig 3:**
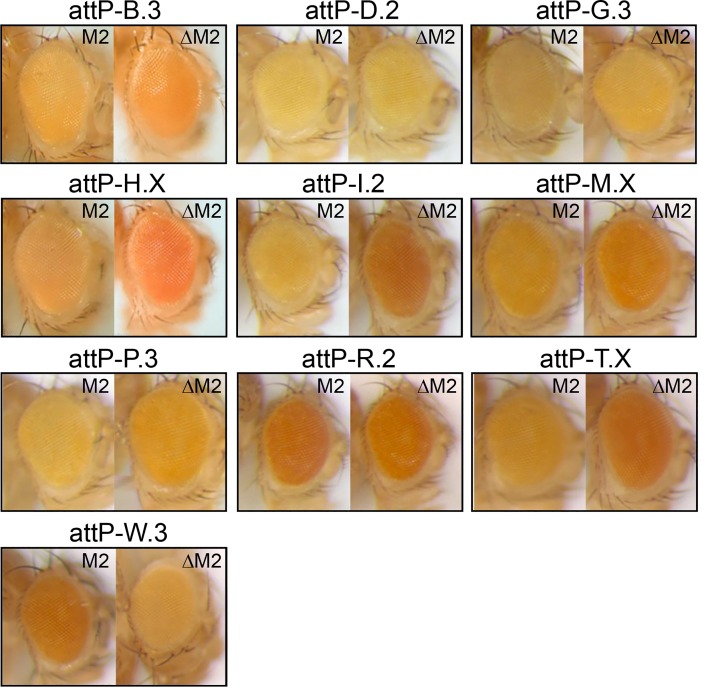
Testing for CPE in flies. Eyes of 2 to 3 day old heterozygous females were photographed before (M2) and after (ΔM2) flipping out the M2 insulator upstream of the mini-*w* gene to test for CPE. Heterozygotes were used to avoid any confounding pairing effects, although CPE is equally apparent in homozygotes. The fly lines shown were used to make *attP* sites. Note that the 3W.3 line shows reverse CPE, suggesting the M2 insulator is blocking a silencer rather than an enhancer.

Using the M2 insulator, 23 viable lines were obtained and tested for CPE. Of these, we used 10 to generate new *attP* landing platforms (Table 1). The *attP* sites were confirmed by genomic sequencing, and their locations were mapped by inverse or TAIL or splinkerette PCR [17–19]. Four of these show clear activating CPE, one shows silencing CPE, and the other five show weak or minimal CPE (Fig 3). In most cases, data on FlyBase indicates that the level of CPE correlates with the expression of nearby genes [20]. For example, three of the four sites showing strong CPE are associated with genes that show high expression in adult eyes (*CG32638*, *Tsp42Ej*, *l(1)G0289*). One of these also shows high expression in third instar larval imaginal discs (*l(1)G0289*). The fourth is associated with a gene with moderate expression in adult eyes and third instar larval imaginal discs (*Actn*).

**Table 1:** Fly lines with *attP* sites and their CPE potential.

Fly line	Chr arm	Genomic site	Orientation	CPE Potential	Integrase gene
attP-B.3	3R	27,281,983	+	++	
attP-D.2	2R	11,912,809	+	+	Yes
attP-G.3	3R	4,433,992	-	++	
attP-H.X	X	13,128,844	-	++++	Yes
attP-I.2	2R	7,040,089	+	++++	Yes
attP-M.X	X	10,366,253	+	+++	Yes
attP-P.3	3R	8,738,659	+	++	
attP-R.2	2R	8,250,415	+	-	
attP-T.X	X	2,037,411	+	+++	Yes
attP-W.3	3L	17,032,287	+	++ (sil)	

The fly line name indicates whether the *attP* site is on chromosome X, 2 or 3. The chromosome arm genomic site is in R6 coordinates. The attP-W.3 line has a silencing CPE (sil). Lines that have been combined with a phiC31 *Integrase* transgene are indicated.

**Fig 4:**
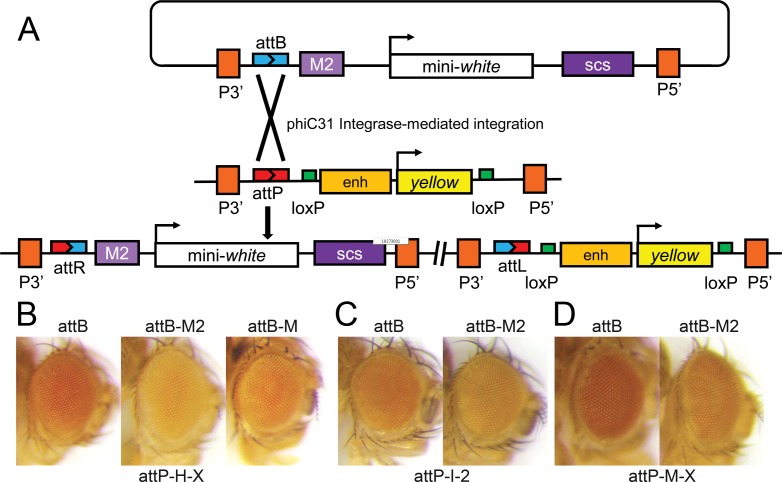
Testing *attP* sites and CPE. (**A**) Integration of the *attB* plasmid into the *attP* docking site results in the test insulator and downstream mini-*w* gene being near genomic DNA. The scs insulator and rest of the plasmid (not shown, indicated by the broken line) is between the mini-*w* and *y*^*+*^ transgenes. One plasmid is shown, although three plasmids were used for integration: one lacking a test insulator, one with a monomer of the M insulator fragment from scs’, and one with the M2 dimer. (**B**) CPE is evident after integration into the attP-H.X line without an insulator upstream of mini-*w*. This CPE is partially blocked by the M insulator and is more effectively blocked when M is dimerized into the M2 insulator. (**C, D**) CPE that can be blocked by M2 is also evident after integration into the attP-I.2 and attP-M.X lines.

To confirm that the *attP* sites are functional, and that CPE could be recapitulated, chromosomes with phiC31 *Integrase* transgenes were introduced into several of the lines. Three of these were used to integrate *attB*-containing plasmids into the *attP* sites. Adjacent to the *attB* site, the plasmids had a mini-*w* gene with a downstream scs insulator. One plasmid had the M2 insulator between *attB* and mini-*w* (Fig 4A), one had a monomer of the M sequence, and one lacked an upstream insulator. These plasmids were derived from pCaSpeR4, so P-element ends were also present. The M fragment is around 215 bp long and has a high affinity BEAF binding site. It had only been tested as a dimer, which functioned as well as a 500 bp scs’ insulator containing a low affinity BEAF binding site in addition to the high affinity site [9]. It was of interest to determine if the monomer with a single BEAF binding site would be as effective as the dimer in blocking CPE.

All three plasmids were integrated into one *attP* line (Fig 4B). Note that additional sequences are present between the scs insulator and the *y*^*+*^ gene compared to the RLY transposon (the P 5’ and 3’ ends and pUC8 sequences), raising the question of whether CPE would be affected. The results clearly show that CPE is observed, and that a monomer of the M sequence is a weaker insulator than a dimer. Therefore only the plasmids lacking an insulator or with M2 were integrated into the other two *attP* lines (Fig 4C and 4D). The results demonstrate that functional *attP* sites were generated in the flies, and CPE that could be blocked by M2 was observed in all cases.

## Discussion

Here we describe the design and use of Dint, a phiC31 disintegrase enzyme for generating *attP* landing platforms in *Drosophila*. The design is based off of a report of reversible phiC31 integration [10]. It is similar to a previously reported tool [11], except ours is based on a nuclear-targeted phiC31 *integrase* transgene with 172 nucleotide changes to better match *Drosophila* codon usage [3]. Use of the *nanos* promoter and 5’ and 3’ UTRs should also improve germline localization in embryos. The design of a P-element based transposon with a mini-*w* reporter gene for detecting CPE that can be blocked by an insulator is also described. We used this transposon with Dint to make *attP* sites in 10 fly lines that show varying degrees of CPE, including one line that had silencing rather than activating CPE. Three lines that showed strong CPE were used to demonstrate that the generated *attP* sites are functional. In all three cases, it was found that the CPE was recapitulated and could be blocked by the M2 insulator. M2 is a dimer of a 215 bp sequence from the 500 bp scs’ insulator [8, 9, 21]. While scs’ has a high affinity and a low affinity BEAF binding site, the M monomer only has the high affinity site. The BEAF binding site is important for the insulator function of M2, since mutating it to eliminate binding by BEAF impairs insulator function [9]. Likewise, M2 insulator function is impaired by the presence of a dominant negative form of BEAF or by a lack of BEAF caused by a null *BEAF* mutation [22, 23]. We found that the M monomer was not as effective as the M2 dimer at insulating against CPE, indicating that two BEAF binding sites make a stronger insulator than a single site. Future work with these lines could further explore the relationship between scs’ sequences and insulator activity.

An advantage of this method is that it allows potential landing locations to be prescreened for expression properties. We did this by random P-element-mediated integration to look for CPE on mini-*w* expression in adult eyes. Different setups could be used, for instance to screen for CPE in embryos or other tissues or to find useful enhancer traps. Once locations of interest are identified, the test transgene can be excised and an *attP* site can be generated so other transgenes can be integrated there.

Before we started, we did not know with certainty where in the genome we would find suitable CPE. In the end we found a strong correlation between CPE and expression levels of nearby genes in adult eyes. High expression of a nearby gene correlated with strong CPE, while low expression of all nearby genes usually correlated with weak or no CPE. With hindsight, this makes sense. Sequence-specific CRISPR/Cas9-directed genome modification can readily be done in *Drosophila* [24]. In principle this could be used to integrate transgenes or *attP* sites at locations chosen purely on high throughput expression data available through FlyBase, or based on some other source of information. This could be a good strategy depending on the quality of the information used to select the integration site. However, the strategy we used provides an unbiased sampling of the genome for finding appropriate integration locations for one’s experimental needs combined with using the highly efficient phiC31 integration system. The set of 10 new *attP* landing platforms that we generated represent a resource that could be useful to members of the fly community.
